# Cancer Resistance to Immunotherapy: Molecular Mechanisms and Tackling Strategies

**DOI:** 10.3390/ijms231810906

**Published:** 2022-09-18

**Authors:** Son Hai Vu, Preethi Vetrivel, Jongmin Kim, Myeong-Sok Lee

**Affiliations:** 1 Institute of Applied Sciences, HUTECH University, 475A Dien Bien Phu St., Ward 25, Binh Thanh District, Ho Chi Minh City 72308, Vietnam; 2 Cellular Heterogeneity Research Center, Department of Biological Science, Sookmyung Women’s University, Seoul 04310, Korea; 3 Department of Pharmacy, National University of Singapore, Singapore 117643, Singapore

**Keywords:** cancer resistance, immunotherapy, mechanism, tackling strategy, gut microbiota

## Abstract

Cancer immunotherapy has fundamentally altered cancer treatment; however, its efficacy is limited to a subset of patients in most clinical settings. The immune system plays a key role in cancer progression from tumor initiation to the metastatic state. Throughout the treatment course, communications between the immune cells in the tumor microenvironment and the immune macroenvironment, as well as interactions between the immune system and cancer cells, are dynamic and constantly evolving. To improve the clinical benefit for patients who do not respond completely to immunotherapy, the molecular mechanisms of resistance to immunotherapy must be elucidated in order to develop effective strategies to overcome resistance. In an attempt to improve and update the current understanding of the molecular mechanisms that hinder immunotherapy, we discuss the molecular mechanisms of cancer resistance to immunotherapy and the available treatment strategies.

## 1. Introduction

Cancer has a negative impact on patients’ health and livelihoods, making it one of the most formidable adversaries of humanity. Cancer immunoediting is a major mechanism for controlling cancer that consists of three sequential phases: elimination, equilibrium, and escape [1]. The interactions between immune systems and cancer cells are dynamic and constantly evolve because of the surrounding living environment, medical interventions, or genetic factors. These factors influence anti-tumor immune responses to varying degrees and at the same time, resistance mechanisms would most likely emerge [2]. The immune system’s role as a major driver of tumor evolution is based on the selective pressure exerted on cells that give rise to tumors [2,3]. Almost every subset of immune cell has been implicated in cancer biology, and because of the complexity of the immune system and tumor heterogeneity, elucidating these cancer cell-immune cell interactions has been a difficult task [4,5]. While traditional methods of cancer therapy such as surgery, radiation and chemotherapy have been shown to improve patient outcomes, advanced cancer remains a major issue. Thus, alternative novel therapeutic approaches are required to achieve complete remission.

Immunotherapy has been widely recognized as a breakthrough treatment for a number of malignancies, earning Drs. James P. Allison and Tasuku Honjo the Nobel Prize in Physiology or Medicine in 2018 for their pioneering work—discovery of a novel cancer therapy by inhibition of the brakes on the immune system [6,7]. Immune-based cancer therapy consists of two major approaches including immune checkpoint blockade (ICB) and adoptive T cell therapy (ACT). Several immune checkpoint inhibitors (ICIs) have been studied extensively [8,9]. ICB is best-known for its remarkable efficacy in treating melanoma, with approximately 20% of recipients experiencing a complete response. More importantly, immune checkpoint therapy also induces a durable complete remission of melanoma [10]. While immune related toxicities induced by PD-1 blockade is similar to those induced by CTLA-4 blockade, they are less common possibly due to PD-1/PD-L1 checkpoint may involve later in T cell response [11]. 

Tumor-infiltrating lymphocyte (TIL) therapy and chimeric antigen receptor (CAR) T-cell therapy are the two types of ACT-based therapy. Cell-based immunotherapy is based on the notion of using the cells of immune system to eliminate cancer [2]. TILs are prepared by isolating cells from surgical samples; the isolated TILs are then cultured to increase the number enough to be transfused back to the patients whose samples were collected. About half of the patients (whose endogenous TILs could be isolated) responded well to immunotherapy and durable complete responses were observed in 22% of advanced melanoma patients, in which 95% of them survived beyond three years [12]. TIL therapy, however, has several major drawbacks, including the requirement for large surgical specimens, tumors with an adequate number of anti-tumor T cells, highly trained personnel, and experienced medical centers. The ACT approach aided by advances in gene-editing technologies and T cell engineering has been developed to address the limitations of the TIL approach [13]. T cell receptors (TCRs) are selectively cloned from TILs to avoid harming normal tissues and to retain the ability to specifically target cancer antigens [2]. Cloned TCRs react to specific cancer antigens that are abundantly expressed in cancer cells but are insignificantly expressed or even not expressed in normal tissue in major histocompatibility complex (MHC)-dependent context [14]. The TCR therapy loses its effectiveness in MHC-downregulated tumors due to a loss of antigen presentation on tumor cells. A wide range of T cell subsets and other immune cells can be transduced with CARs, which are recombinant cell surface receptors for tumor antigen redirecting the specificity and function of the targeted cells. Fundamentally, CARs enable over-riding tolerance to self-antigens and excel physiological antigen recognition ability of immune cells to modulate T cell expansion and persistence, as well as T cell and NK cell activation to target cancer [15]. The CAR T approach is established based on the expansion of T cells with engineered surface molecules such as CD28 and 4-1BB [16,17]. In the famous CAR T-related clinical trial in one of the hematological malignancies [18], the results were remarkable with 90% of the patients achieved complete remission. Although the severe cytokine-release syndrome (indicated by elevation of IL-6 levels after treatment) was observed in all the patients, it was well-controlled using treatment with tocilizumab (IL-6R blocking antibody). This clinical trial has paved the way for the use of CAR T therapy in the treatment of other malignancies and solid tumors. A mutual feature of TCRs and CAR T therapy is the expansion of genetically engineered T cells toward specific antigen targets [19,20,21]. Although CAR T therapy demonstrates remarkable efficacy in patients with hematological malignancies, the outcomes are limited by the tumor microenvironment (TME), particularly in solid tumors [22]. However, by repurposing IL-9R signaling using a chimeric orthogonal cytokine receptor, T cells gain new functions including characteristics of stem cell memory and effector T cells leading to augmented anti-tumor activity against solid tumors [23]. CAR-NK cell therapy has started to gain attraction as an alternative to CAR-T approach. This NK-based therapy possesses certain advantages compared to CAR-T approach such as shortened production time, recognition and exerting lethal attack on tumor cells by NK cell native receptors, unlimited use of allogeneic NK source without concern of graft versus host diseases, and the potential of using NK cell line or iPSC-NK to generate ready-to-use treatment [24]. However, CAR-NK therapy also has several need-to-improve points, including lack of in vivo persistence and sensitivity to free-thaw process leading to loss of viability and/or activity [24].

**Figure 1:**
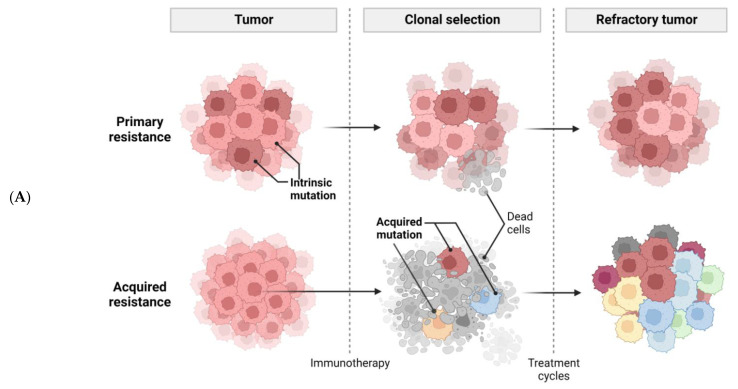

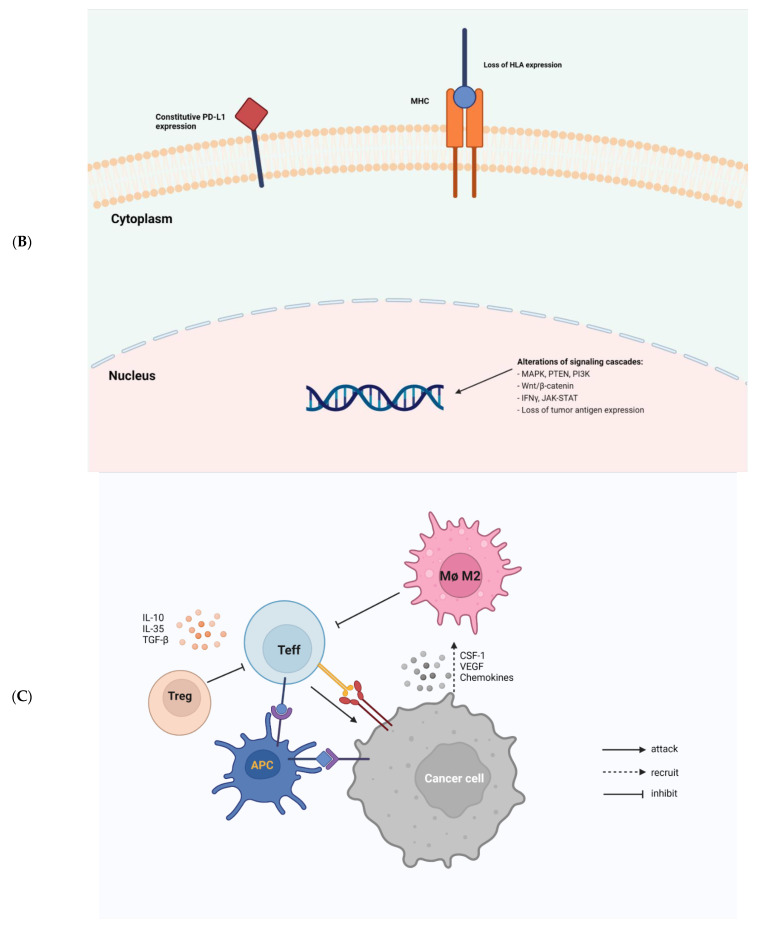
Tumor-intrinsic and -extrinsic mechanisms of resistance to immunotherapy. (**A**), a general diagram of how cancer resistance emerges upon immunotherapeutic treatment. Intrinsic mutation in tumor cells renders cancer cells primary resistance upon immunotherapy leading to refractory tumor. Acquired resistance is generated in survived cancer cells upon immunotherapy leading to refractory tumor. (**B**), tumor-intrinsic mechanisms of resistance. Intrinsic factors include constitutive PD-L1 expression, loss of HLA expression in cancer cell membrane, and alterations of signaling cascades such as MAPK, PTEN, PI3K, Wnt/β-catenin, IFNγ, JAK-STAT, and loss of tumor antigen expression. (**C**), tumor-extrinsic mechanisms of resistance. The complex interplay of immune cells and cancer cells in tumor-microenvironment. Cancer cells are constantly subjected to bombardment by immune cells such as NKs and Teffs. However, cancer cells produce CSF-1, VEGF, and several chemokines to recruit M2 macrophages which in turn inhibit Teffs. Treg also induce cytokines, such as IL-10, IL-35, TGF-β to impede Teff responses.

Despite promising results in patients administered with immunotherapy, the majority of patients did not respond completely, due to primary resistance. Even with some patients who responded, recurrence may have occurred over time due to acquired resistance. Two major mechanisms (Figure 1A), coping strategies, and perspectives will be further discussed.

## 2. Molecular Mechanisms

### 2.1. Tumor Intrinsic Factors of Resistance to Immunotherapy

Cancer cells employ sophisticated strategies such as avoiding immune recognition and inducing immunosuppressive TME to evade immune attack. These mechanisms may occur before (primary resistance) or after (adaptive resistance) immunotherapy. Several important tumor-intrinsic pathways are associated with primary and adaptive resistance including (1) the mitogen-activated protein kinase (MAPK) pathway and/or loss of phosphatase and tensin homolog (PTEN) expression, which enhances PI3K signaling, (2) expression of WNT/β-catenin signaling pathway, (3) loss of interferon-gamma (IFNγ) signaling pathways, and (4) loss of tumor antigen expression (Figure 1B) [25].

In cancer, VEGF and IL-8 are induced by the MAPK cascade to inhibit the recruitment and functions of T cells [26]. Inhibition of MAPK signaling combined with PD-1/PD-L1-targeted or BRAF-targeted therapies leads to enhanced anti-tumor immune responses such as the increased presence of TILs [27,28,29]. Resistance to ICI can also be caused by the loss of PTEN, which promotes PI3K signaling to enhance proliferation of malignant cells [30]. PTEN loss in melanoma is associated with decreased gene expression of IFNγ and granzyme B in immune cells, as well as the infiltration of CD8^+^ T cells. PTEN deletions and mutations are more common in non-T-cell-inflamed tumors than in T-cell-inflamed tumors. As a result, ACT was less effective in treating PTEN-deficient tumors than PTEN-expressing tumors in mice model [30]. Moreover, a significant enrichment of somatic PTEN mutations was associated with resistance to ICIs, probably contributing to the immunosuppression environment in non-responders with glioblastomas [31].

The observation in many human cancers of an aberrant activation in the Wnt/β-catenin signaling has prompted the use of Wnt signaling inhibitors in cancer treatment. Constitutive Wnt signaling via β-catenin stabilization can induce T cell exclusion from the vicinity of cancer cells, impairing anti-tumor immunity and promoting immunotherapy resistance [32]. Experimental data from mice model suggest that tumors with elevated β-catenin levels have a loss of CD103^+^ DCs due to decreased CCL4 expression. Furthermore, ICB is more effective in targeting β-catenin loss tumors than β-catenin-expressing tumors. In the same vein, β-catenin signaling-related genes are higher expressed in non-T-cell-inflamed tumors [32]. Results from the clinical study by Luke et al. [33] strongly support the rationale of targeting Wnt/β-catenin pathway in cancer. Additionally, mutations in β-catenin signaling molecules showed a significant enrichment in non-T-cell-inflamed tumors, and moreover, activation of Wnt/β-catenin signaling was found in 90% of examined non-T-cell-inflamed tumors [33]. 

The IFNγ signaling pathway, which functions through JAK-STAT signaling [34], has dual effects on anti-tumor immunity. Tumor-specific T-cell-induced IFNγ can induce lethal anti-tumor immunity by promoting tumor antigen presentation, recruiting of other immune cells, and directing anti-proliferative and pro-apoptotic effects toward the tumor cells [35,36]. On the other hand, cancer cells can downregulate or mutate molecules involved in the IFNγ signaling cascade or harness the immunosuppressive functions of IFNγ to evade destructive immunity [34,37]. Indeed, an increased enrichment of mutated IFNγ signaling related genes such as interferon gamma receptor 1 and 2, Janus kinase 2, and interferon regulatory factor 1 was observed in non-responders to ipilimumab (anti-CTLA-4 mAb) recapitulating the loss of IFNγ signaling-related genes in cancer cells is a mechanism of resistance to anti-CTLA-4 therapy [38]. Patients with any of these mutations are likely to be resistant anti-PD-1 therapy due to a lack of PD-L1 expression upon IFNγ exposure [39]. MHC-I molecules (also known as human leukocyte antigen or HLA) bind peptides derived from proteins produced in cells and transport them to the cell surface to display antigenic information. It enables CD8^+^ T cells to identify pathological cells that synthesize abnormal proteins, such as cancer cells that express mutated proteins [40]. The absence or acquired poor molecule expression of HLA allows neoantigens to remain undetected by the immune system. Defects in the antigen presentation pathway are caused by a number of mechanisms, including genetic and epigenetic alterations [41]. For instance, defects in the MHC-I antigen presentation pathway are the primary mechanism for the loss of tumor-specific MHC-I expression. Downregulated TAP1/TAP2 which results in defective peptide presentation leads to decreased MHC-I stability and its expression on the tumor surface [42]. 

Furthermore, impaired antigen presentation in tumors is caused by structural changes of the MHC-I complex, notably including loss of heterozygosity associated with chromosome 6p21 and from the poor expression of β2M [42]. The loss of key transcription factors, such as NF-κB and NLRC5, and epigenetic alterations (such as DNA hypermethylation and downregulation of histone deacetylases) can affect the transcription of MHC-I pathway genes, thereby contributing to immune evasion of cancer cells [42]. Downregulation of the NLRC5 transcription factor is associated with decreased expression of target genes such as MHC-I, ß2M, TAP, and immunoproteasome subunits in many cancers, including prostate, lung, uterine, melanoma, and thyroid [43]. Interferons are another type of signaling molecule that can induce MHC-I expression [44]. The activation of signal transducer and transcription proteins (STAT1, 2, 3) due to type I and type II interferon signaling upregulates MHC-I expression [44]. As the function of IFNs in antigen processing and presentation pathway is indispensable, the impairment of IFN signaling leads to the downregulation of MHC-I, implying that this molecular crosstalk has a significant impact on tumorigenesis. Additionally, upregulation of microRNAs (miRNA) inhibits MHC-I expression in several types of cancer including melanoma, esophageal carcinoma, and colorectal cancer. These non-coding RNAs are also found to regulate multiple signaling molecules of antigen presentation pathway, such as TAP1/TAP2 and calreticulin [42]. MHC-I expression abnormalities are classified as irreversible or reversible based on the ability to restore molecule expression with cytokine or pharmaceutical treatment [45]. MHC-I downregulation is frequently associated with reduced TILs which may correlate with poor clinical outcomes in many cancers, including melanoma, glioblastoma, colorectal, bladder, uterine, cervical, head/neck, breast, and others [46]. As a result, the MHC-I downregulation may be used as a prognostic factor for immunotherapy. Thus, understanding pathogenesis by determining whether there are potential ways to restore MHC-I expression can augment the immune system to control malignancies. 

The role of epigenetic changes in cancer cells is still debated. On the one hand, these modifications affect antigen processing, presentation, and immune evasion through changes in the expression of immune-related genes, suggesting that using demethylation agents may have therapeutic implications [47,48]. For example, T helper cells with demethylated DNA could be used as antigen presenting cells (APCs) to generate cytotoxic T lymphocytes (CTLs) and natural killer cells to target cancers [49]. On the other hand, the loss of DNA methylation as a result of cell proliferation accompanied by high mutation and copy number load can promote tumor immune evasion [50]. TSA, a histone deacetylase inhibitor (HDCAi), promotes the expansion of a population of myeloid-derived suppressor cells (CD11b+Ly6C+F4/80intCD115+), implying that acetylation influences myeloid cell differentiation [51]. In contrast, Wang and colleagues reported that using HDACi SAHA reduces MDSC accumulation in the spleen, blood, and tumor bed. Furthermore, exposure of bone marrow cells to SAHA eliminates GM-CSF-induced MDSC population through increased intracellular ROS [52]. 

### 2.2. Tumor Extrinsic Factors of Resistance to Immunotherapy

The presence of immune cells in human tumors implicates the mixed role of immune cells in tumor growth and progression (Figure 1C). Macrophages, myeloid-derived suppressor cells (MDSCs), regulatory T cells (Tregs), effector T cells (Teffs), DCs, natural killer cells (NKs), and B cells are among the most common immune cells in the TME. Each immune cell subset contributes to pro- or anti-tumor immunity with distinct mechanisms.

Tregs are an important subset of T cells that helps to prevent excessive immune responses and autoimmunity. Tregs can infiltrate human tumors and promote tumor growth [53]. These FoxP3-expressing cells either directly impede Teff responses by physical contact, or indirectly by suppressing the latter via the secretion of inhibitory cytokines, including IL-10, IL-35, and TGF-β [54,55,56,57]. Upon anti-CTLA-4 mAb treatment, the ratio of Teffs to Tregs was positively associated with treatment response, which is dependent on the presence of Fcγ receptor-expressing macrophages [58,59], suggesting the use of anti-CTLA-4 antibodies with enhanced FcγR binding profiles to achieve robust anti-tumor responses and improved survival [60]. In a clinical trial using ipilimumab to treat patients with advanced melanoma, increased TILs were found to be associated with better outcomes [61]. A clinical follow-up study demonstrated that while anti-CTLA-4 immunotherapy promoted intra-tumoral Teff infiltration, it did not cause FoxP3^+^ T cell depletion in human cancers [62]. These pioneering studies on the balance between Teffs and Tregs suggest that an increased number of tumor-infiltrating Teffs, rather than the depletion of Tregs may be used to predict sensitivity to anti-CTLA-4 immunotherapy. If the ratio of these two T cell subsets within the TME in response to immunotherapy does not favor Teffs, resistance is likely to occur throughout the course of treatment.

MDSCs comprise a group of neutrophils and monocytes with potent immunosuppressive properties that may mediate immune responses elicited by T cells, B cells and NK cells [63]. Human MDSCs express markers such as CD11b^+^ and CD33^+^; however, other types of MDSCs include the presence of HLA-DR, CD33, and CD15 [64]. Due to their important roles in promoting angiogenesis, tumor invasion, and metastasis, these cells have emerged as therapeutic targets in cancer [63,65,66,67]. A high intra-tumoral number of neutrophils has shown a negative correlation with clinical outcomes in patients with cancer [68]. Indeed, Si et al. [69] using multidimensional imaging, provided direct evidence that MDSCs inhibit the expression of Teff-secreted Granzyme B and Ki67 (markers for cytotoxicity and proliferation of Teff, respectively). The presence of MDSCs in the TME is closely associated with the efficacy of immunotherapy, as blockade of these cells leads to improved pre-clinical [70] and clinical outcomes [71]. Kaneda et al. [72] suggested that the macrophage PI3Kγ is a critical molecular switch that controls immune suppression by inhibiting NF-κB activation and stimulation of C/EBPβ activation, which are AKT- and mTOR-dependent, to promote immune suppression. More importantly, targeting cancer cells by a combination of selective inactivation of macrophage PI3Kγ and ICIs could overcome cancer resistance to checkpoint blockade therapies. In an ICB resistance setting in mice, to overcome resistance, the molecularly selective pharmacological targeting of the gamma isoform of PI3K in myeloid cells restored the sensitivity of immune checkpoint blocking antibodies [73].

Tumor associated macrophages (TAMs) are also associated with patient responses to immunotherapy [74,75], with the M1 subtype promoting anti-tumor immunity and the M2 subtype promoting tumorigenesis. These cells are categorized according to their distinct activation pathways and expression of surface molecule. Recruitment of TAMs to tumor sites is mediated by tumor-derived effector proteins such as CSF-1, VEGF, and chemokines [76]. CSF1/CSF1R axis is crucial for the recruitment of TAMs; therefore, targeting CSF1/CSF1R signaling may have synergistic effects with immunotherapy in suppressing refractory tumors [77,78,79,80]. A higher density of TAMs is correlated with poor clinical prognosis in cancer patients [81,82]. In a mouse model of lung adenocarcinoma, Fritz et al. [83] found that the depletion of TAMs may reduce tumor growth due to the downregulation of M2 macrophages. It has been proposed that the inactivation of CCL2 and/or CCR2 signaling is attributed to this phenomenon. In the same vein, similar findings were obtained in other cancer types such as T cell lymphoma [84], colon cancer [85], lung cancer, and breast cancer [85,86,87], suggesting that the use of pharmacological or biological strategies to inhibit or eliminate these macrophages in the TME may improve patients’ outcome. 

Cancer causes chronic inflammation, which depending on the context, may support tumor development [88]. Hence, anti-tumor responses and signals may also upregulate inhibitory genes and signaling pathways such as IFNγ, CTLA-4, and PD-L1 in immune cells. Activation of T cell via TCR signaling and CD28 co-stimulation increases CTLA-4 expression [6]. Increased IFNγ production by Teffs results in increased production of PD-L1 in both cancer cells and immune cells to hinder anti-tumor responses [2]. The production of several immunosuppressive molecules, including indolaimine-2, 3-deoxygenase, and CEACAM1, is induced by the pro-inflammatory IFNγ leading to peripheral tolerance, an inhibition of NK-mediated cytotoxicity and impaired effector T cell function [89,90]. Both malignant cells and phagocytic cells can produce immunosuppressive cytokines that inhibit local anti-tumor immunity. For example, the immunosuppressive TGF-β stimulates Tregs to regulate angiogenesis and immune responses [91]. Moreover, poor prognosis was associated with elevated TGF-β levels in several malignancies [92]. Clinical studies have suggested that combining ICI and selective inhibition of TGF-β receptor and that combining radiation therapy with TGF-β inhibition synergistically improve anti-tumor responses [93,94]. Multiple chemokines and their respective receptors are essential for the migration of immunosuppressive cells including MDSCs and Tregs to tumor sites [95]. Tumor cells produce multiple chemokines such as CCL2, CCL5, CCL7, and CXCL8, which bind to MDSCs CCR1, CCR2, or CXCR2 to recruit MDSCs to the TME [96,97]. Inhibiting these chemokine receptors or targeting them in combination with ICB may reduce immune evasion and promote anti-tumor T cell responses [98,99,100]. 

Cancer-associated fibroblasts (CAFs) are a diverse stromal cell population with multiple functions such as matrix deposition and remodeling, crosstalk with infiltrating leukocytes and reciprocal interactions with cancer cells [101]. The existence and the role of activated CAFs in the microenvironment are linked to a poor prognosis in various cancers [102]. CAFs have been implicated in influencing the function of various immune cells toward an immunosuppressive phenotype via multiple mechanisms, in addition to their ability to recruit immune cells that promote tumor growth. Notably, a recent understanding of CAF heterogeneity in origin and function suggests that driving immune suppression may be mediated by distinct subpopulations of CAFs. For example, mesenchymal stromal cells (MSCs) derived from bone marrow are a significant source of CAFs in breast cancer [103]. Surprisingly, MSCs also mediate immunosuppression in the physiological wound healing process. Pro-inflammatory cytokines (IFN, TNF, or IL-1) induce MSCs to produce iNOS, suppressing T cell function in acute liver injury models [104]. Tumors may alter the physiological functions of MSCs and fibroblasts to induce the formation of an immunosuppressive TME via the effect of CAFs on specific immune cell populations [105]. T-cell exclusion plays a vital role in shaping the low response rate to immunotherapy in CAF-rich tumors [106]. Different immunotherapy modalities such as anticancer vaccination, and anti-PD-1 allow the heterogeneous cell population to suppress the response by excluding CD8^+^ T cells but not CD4^+^ T cells or macrophages from tumors. T-cell exclusion is associated with increased CTLA-4 expression, which can be inhibited to overcome lymphocyte exclusion [106]. Thus, identifying and targeting novel resistance mechanisms such as CAF-related signaling would improve cancer therapies. However, CAFs research is particularly challenging to translate into clinical benefit as CAFs can either promote or inhibit tumorigenesis [101].

Other more complex factors, such as age, gender, pre-existing conditions, and intestinal flora, may also contribute toward cancer resistance to immunotherapy. Aging is frequently accompanied by deteriorating immunity and various health-related issues, thus there is a perception that the efficacy of anti-cancer therapeutic approaches may also decline with age. However, in a meta-analysis of randomized controlled trials with ICB, age was not determined to be a factor in how patients responded to the therapy [107]. Systemic immunity is influenced by sex and gender. Women are known to mount more robust immune responses than men [104]. Another meta-analysis of randomized controlled trials of immune checkpoint therapy for melanoma and human non-small cell lung carcinoma (NSCLC) also revealed that the magnitude of benefit is sex-dependent, with women having a lower pooled hazard ratio for overall survival than men [105]. There is also a knowledge gap regarding how immunotherapy affects the anti-tumor immune response of cancer patients with pre-existing common health conditions such as diabetes, obesity, and hypertension. The benefits of immunotherapy to cancer patients with obesity are ambiguous [108]. Diabetes has negative effects on ICB in metastatic NSCLC [109]. Mechanistic studies are needed to shed light on the linkage between metabolic pathologies and immunotherapy efficacy.

Gut microbiota have been extensively studied over the last two decades and the role of microbes residing in the human gut has had an impact far beyond infectious diseases. Changes in the gut microbiome have been linked to various metabolic disorders and cancers [110]. Because the adaptive immune system shapes the gut flora and vice versa, immunotherapy-responsive cancer patients may have a different gut microbiome composition than non-responders [111]. Two pioneering studies on gut microbiome in cancer settings have laid the foundation for the concept that gut microbiota modulates immunotherapy [112,113]. In murine models, Vétizou et al. [112] found that optimal responses to CTLA-4 blockade rely on *Bacteroides* spp. Oral supplementation with *B. fragilis*, or its derived polysaccharides, or using *B. fragilis*-specific ACT-based approach restored the efficacy of anti-CTLA-4 therapy. Commensal *Bifidobacterium* in the gut flora may modulate anti-tumor immune responses and facilitate PD-L1 blockade’s efficacy [113]. Subsequent studies have further characterized the association between human gut microbiota and the outcome of cancer patients treated with immune checkpoint inhibitors [114,115,116]. Upon PD-1 immunotherapy in melanoma patients, enhanced systemic and anti-tumor immunity was reported in responders with favorable gut microbiome, such as the abundance of bacteria of the *Ruminococcaceae* family [114]. Similarly, a clear linkage was found between commensal microbial composition and clinical response in metastatic melanoma patients treated with anti-PD1 therapy [116]. *B. longum*, *Collinsella aerofaciens*, and *Enterococcus faecium* were among the most abundant bacteria present in the gut flora of responding patients. Fecal transplantation from responders to mice increased T cell responses, higher efficacy of ICB, and improved immune-mediated tumor control [116]. In patients with lung and kidney cancers, Routy et al. [115] found that antibiotic consumption was negatively associated with anti-PD-1 immunotherapy, and low levels of *Akkermansia muciniphila* were found in non-responders. Interestingly, this defect was reversed by oral gavage with the bacterium, which is mechanistically via increased recruiting CCR9^+^CXCR3^+^CD4^+^ T cells into tumor beds in an IL-12 dependent manner [115]. Many of the molecular mechanisms that affect host response to immunotherapy have not been fully characterized, but some are already underway. The enterococci, *E. faecalis*, could augment immunotherapy efficacy by expressing and secreting the peptidoglycan hydrolase secreted antigen A (SagA) which generates immune-active muropeptides to activate NOD2-dependent NF-κB- and MAPK-mediated signaling of pro-inflammatory genes [117]. 

## 3. Tackling Strategies

Therapeutic resistance in immunotherapy remains a major concern, and efforts are needed to identify biomarkers that can be used to monitor and overcome resistance. A gold standard predictive biomarker is elusive due to the complexity of tumor heterogeneity and anti-tumor immunity which vary from patient to patient [118,119]. While tumor mutational load and markers of immune infiltrate within a tumor are important predictive biomarkers, none of these markers can track intrinsic and acquired resistance individually. Another monitoring strategy is the evaluation of longitudinal tumor specimens. Unlike traditional approaches, this strategy can assess tumor dynamics during treatment and identify predictive biomarkers [120]. Therefore, a combination of pre-treatment and post-treatment biomarkers is required to accurately assess the effectiveness of immunotherapy in recipients. 

As ICI response rates are low in recipients with “cold” tumors (characterized by the lack of T-cell infiltration), turning “cold” tumors to “hot” tumors is one of the potential strategies to prevail the resistance [121,122,123]. To that end, a complete understanding of the process of driving T cells into tumors in which various factors take part in managing the cellular movement will aid in the development of more effective and safe immunotherapies. Combinatorial therapies with the core blocking immune checkpoint(s) have been tested to select the best strategy to overcome resistance (Table 1). 

Several combination strategies have shown promising results in clinical trials. For example, long-term overall survival was significantly increased in patients with recurrent glioblastoma that were subjected to preoperative injection of ipilimumab and nivolumab (anti-PD-1 mAb) following safe resection of recurrent glioblastoma [168]. Pembrolizumab (anti-PD-1) plus V937 (Coxsackievirus A21) was manageably tolerated in patients with advanced melanoma resulting in clinical benefit [169]. The combination of molecularly targeted therapy and immunotherapy may serve as a potentially effective treatment owing to its favorable effect on anti-tumor immunity and synergistic effect when combined with immune checkpoint blockade. This type of treatment has been intensively tested; however, hepatotoxicity was deemed to be the main concern in studied patients [170,171]. Increased histamine receptor H1 (HRH1) expression is found in the TME and induces T cell dysfunction via polarizing macrophages toward an M2 phenotype, suggesting HRH1 as a potential target for immunotherapy. Indeed, anti-PD-1/PD-L1 treatment has synergistic effects with HRH1-specific antihistamines (H1-antihistamines) reducing the death rate of melanoma, lung, breast, and colon cancer patients [172].

In contrast to conventional killer T cells, which are APC-dependent and not capable of using innate receptors in response to stress, killer innate-like T cells (ILTCKs) have innate immune cell-like behavior and are APC-independent, allowing them to prepare for lethal attack. Since they are reprogrammed during development, ILTCKs can recognize unmutated antigen and do not cause autoimmunity [139]. Their expansion and effector differentiation are dependent on IL-15, which is produced by many cancer cells; interestingly, inducible activation of IL-15 signaling in adoptively transferred ILTCK progenitors suppresses tumor growth while deletion of IL-15 in cancer cells increases tumor growth. The T cell subset is far more durable than typical killer T cells because PD-1 is not produced in by ILTCKs making them a potential target for immune cell-based therapy [173].

Both type I and II IFN signaling are important to anti-tumor immune responses as IFNs are required for antigen recognition and the coordination between adaptive and innate immune cells [174]. As a result, changes in the IFN signaling cascade including loss-of-function mutations and genomic alterations are correlated with ICB resistance [38,39,175]. Minn group has elegantly characterized the role of IFNγ signaling in resistance to ICB. Tumors may acquire STAT1-related epigenomic modifications due to prolonged IFNγ signaling that promotes IFN-stimulated genes (ISGs) and ligands for T cell inhibitory receptors [37]. Thus, targeting IFN signaling that involves the resistance program renders ICB-resistant tumors responsive to a single ICB therapy. Moreover, blockade of IFNγ signaling suppresses the expression of ISGs (common biomarkers for ICB response) in cancer cells while increases ISGs in immune cells [174]. This higher immune vs. cancer ISGs debilitate inhibitory pathways allowing NK/ILC1s (capable of killing tumors) to mature. In poor MHC-I or neoantigen tumors, IFNγ is utilized by exhausted T cells to drive maturation of PD1^+^TRAIL^+^ ILC1 cells that eliminate the tumors [174].

Manipulation of the gut microbiota to overcome resistance to immunotherapy has received increasing attention. The rationale for this is strongly supported by elegant studies discussed previously [114,115,116]. Thus, fecal microbiota transplant (FMT) has been used to overcome resistance to anti-PD-1 therapy in melanoma patients [176,177]. Findings from Zarour group indicated that 40% of recipients (6 out of 15) received clinical benefits from the combination of anti-PD-1 and FMT, which was well-tolerated and induced quick and prolonged microbiota perturbation. The increased abundance of commensal bacteria associated with anti-PD-1 blockade, promoted CD8^+^ T cell activation, and reduced frequency of immunosuppressive interleukin-8–expressing CD14^+^ myeloid cells were found in responders to the combination therapy [177]. Baruch et al., demonstrated the efficacy of a therapeutic combination using a similar strategy to overcome resistance to anti-PD-1 therapy. Clinical responses were observed in three recipients (30%), including one complete response [176]. Because of the complexities of the gut microbiome and its interactions with other biological systems, identifying specific response-inducer microbiota characteristics is challenging. There is still room for improvement of the FMT-based therapy since more than half of the immunotherapy-refractory patients did not respond to the therapeutic combination in the two clinical trials.

Desired nanomaterials should be highly biocompatible and non-toxic. Once developed, these nanoscale particles are simple to manipulate, and possess favorable biological features for combating cancers including flexibility in biodistribution, specific targeting, bioavailability, and enhancing immunogenicity. Due to intensive research efforts, the effectiveness of immunotherapy has been improved toward a more patient-friendly and targeted approach [178]. The translational gap between animal and human studies is the main reason for the relatively limited availability of nanomedicine. Nanotechnology-based immunotherapy has achieved certain promising outcomes in various cancers in pre-clinical models [178]. The combination of a stimulator of an interferon gene (STING) agonist-loaded lipid nanoparticles (STING-LNP) and anti-PD-1 monotherapy synergistically enhanced anti-tumor activity through NK cell activation, while anti-PD-1 therapy alone failed to limit lung metastasis of melanoma in mice [179]. STING agonists have been used to provide clinical benefits; however, inefficient cytosolic entry and systemic toxicity are the main concerns of this strategy. To overcome these limitations, the microbubble-assisted ultrasound-guided immunotherapy of cancer (MUSIC) platform was developed, which inhibited tumor growth by bridging innate and adaptive immune responses in murine models [180]. Specifically, the nanocomplex-conjugated microbubbles that target APCs deliver cyclic guanosine monophosphate-adenosine monophosphate (cGAMP) into the cytosol, where they induce a robust activation of STING and IFN regulatory factor 3 (IRF3) and activate downstream NF-κB signaling pathway. This would stimulate APCs that subsequently prime CTLs. MUSIC can further sensitize poorly immunogenic tumors to PD-1 blockade by enhancing anti-tumor responses [180].

Another example of the implication of NPs in promoting response to checkpoint inhibitor therapeutics is reported by Bhatia group; their engineered nanoparticles carrying immunostimulatory oligonucleotides plus anti-CTLA-4 treatment achieved synergistic tumor suppression in several animal models of various cancers [181]. However, the mechanism underlying this synergistic effect remains to be elucidated. A nanoconjugate comprising hollow manganese (H-MnO_2_) was developed to assist TME-specific imaging, which elicited a remarkable synergistic therapeutic effect by inducing anti-tumor immune responses, such as the presence of immune cells at tumor sites and cytokine levels and relieving hypoxic condition by decomposing endogenous H_2_O_2_ [182]. The elegant hollow structure may prevent degradation of drug payload from the acidic TME and allow the co-loading of a photodynamic agent, chlorine e6, with the anti-cancer drug, doxorubicin. Furthermore, this platform combined with immune checkpoint blockade has resulted in a significant reduction in both primary and distant tumors [182]. Potent and prolonged cellular immunity against tumor cells has also been observed in another nanoconjugate platform [183]. A subunit vaccine comprising NPs coupled with CpG-B or CpG-C oligonucleotides, co-delivered with low-dose adjuvant (4 µg) and antigen (prepared on separate NPs) to limit toxicity and target the lymph node induced enhanced maturation of dendritic cells, and Th1-derived cytokine secretion, leading to robust activation of CD8^+^ T cells with strong memory recall [183]. One of the major challenges in ACT therapy is the immunosuppressive TME. NPs, fortunately, can alleviate the issue to a certain extent. Employing poly(lactic-co-glycolic acid) (PLGA) NPs indirectly improves manufacturing of T cells by enhancing antigen presentation by DCs in the absence of TLR ligands [184]. Protein antigens encapsulated in the developed nanoplatform may stimulate DC to induce strong immune responses via the activation of CD4^+^ and CD8^+^ T cells leading to a complete regression of planted tumors [184]. In addition, DNA-carrying nanoparticles may be used to introduce leukemia-targeting CAR genes into T-cell nuclei, resulting in long-term cancer remission [185]. 

## 4. Perspectives

Cancer is a systemic disease, and the entire immune system constantly changes in the presence of cancer [186]. Immunotherapy induces new immune responses rather than working on pre-existing ones. An in-depth understanding of tumor-intrinsic and tumor-extrinsic factors involved in cancer immunotherapy resistance necessitates research across tumor types, patient populations and therapies. High-throughput, high-dimensional, single-cell technologies have resulted in ground-breaking discoveries and atlases of various TMEs, potentially leading to the development of more effective immunotherapies and personalized medicines. However, mechanistic studies on the interaction between immune cells and cancer cells has primarily focused on the TME, with less emphasis on the regulatory role of peripheral immune cells in tumor biology. Therefore, not only the TME but also the global immune macroenvironment must be taken into account [5]. Non-responders typically have a disrupted immune system, thus, restoring a compromised immune system to an active and healthy homeostatic immune set point will benefit patients and may enhance the effectiveness of immunotherapy.

**Figure 2:**
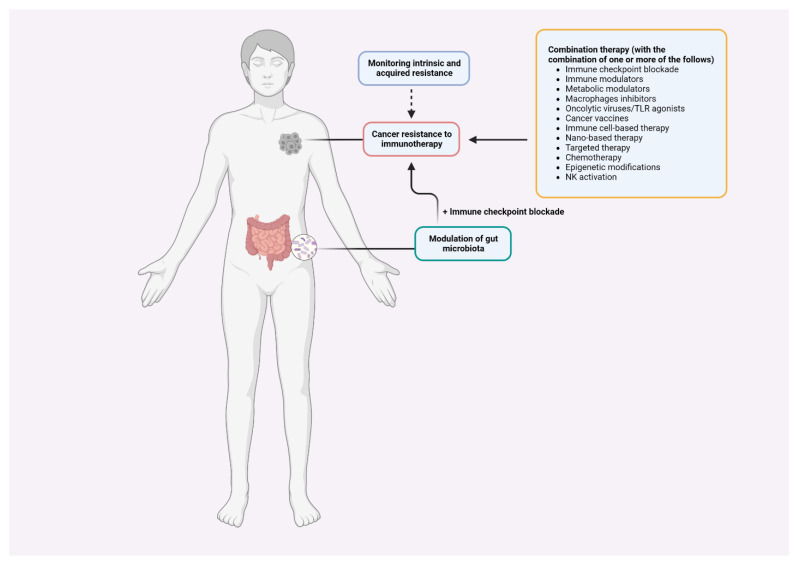
Tackling strategies. To tackle resistance effectively, tumors should be monitored followed by a number of therapeutic strategies such as combination therapy (see Table 1) and modulation of gut microbiota plus immune checkpoint blockade.

Considering that the majority of patients with a certain type of cancer either do not respond or do not fully respond to immunotherapy, bringing clinical benefit to these patients necessitates a thorough understanding of the underlying mechanisms. This critical understanding would result in an effective anti-tumor response and reveal new tumor-intrinsic and -extrinsic factors that contribute to primary and adaptive resistance to immunotherapy for molecular targeting (Figure 2). T cells have been effectively and widely used in the context of cancer immunotherapy. Although T cell immunity has yielded impressive mechanistic insights, further research into the regulation and interaction of T cells with other immune cells, such as APCs and NK cells, is required to advance T cell-based immunotherapy. Breakthrough findings indicate that T cell-mediated anti-tumor immunity is augmented through modulation of commensal bacteria species in the gut microbiome. This may lead to the development of another innovative approach to improve patients’ responses to immunotherapy, one that combines the ability of other commensal bacteria to regulate anti-tumor immune responses with combination strategies. Thus, a comprehensive understanding of how the complex gut microbiota elicit anti-tumor immunity in both pre-clinical and clinical settings, as well as how immunotherapy shapes the gut microbiome would broaden the current knowledge to improve efficacy and overcome resistance. Diet, lifestyle, stress, environment, and genetics are among the numerous factors that can modulate the composition of the human gut microbiome to affect immunotherapy response [117,187]. Well-designed experiments and human trials are essential for improving the current understanding and effectiveness of immunotherapy, especially in patients who have developed resistance to ICB therapy. NP-based immunotherapy is gaining popularity as a powerful treatment to promote favorable immune responses and effectiveness. However, major challenges, particularly the gap between pre-clinical outcomes and clinical applications, must be carefully addressed. Studies related to NP-based immunotherapy are urgently needed to pave the way for clinical translation before other factors are considered. 

## Figures and Tables

**Table 1:** Proposed combinatorial therapies to overcome resistance to cancer immunotherapy.

No.	Approach	References
1.	Combination checkpoint blockade	[124,125,126]
2.	Checkpoint blockade plus metabolic modulators	[127,128]
3.	Checkpoint blockade plus other immune modulators	[129,130,131,132,133,134,135]
4.	Checkpoint blockade plus macrophage inhibitors	[136,137]
5.	Checkpoint blockade plus oncolytic viruses or plus TLR agonists	[138,139,140]
6.	Checkpoint blockade plus cancer vaccines	[141,142,143,144]
7.	Checkpoint blockade plus ACT	[145,146]
8.	Checkpoint blockade plus targeted therapies	[147,148,149,150,151,152,153,154]
9.	Checkpoint blockade plus radiation therapy	[155,156,157,158]
10.	Checkpoint blockade plus chemotherapy	[159,160,161,162,163]
11.	Checkpoint blockade plus epigenetic modifications	[164,165]
12.	Checkpoint blockade plus NK activation	[166,167]

## References

[1] Dunn G.P., Bruce A.T., Ikeda H., Old L.J., Schreiber R.D. (2002). Cancer immunoediting: From immunosurveillance to tumor escape. Nature Immunol..

[2] Sharma P., Hu-Lieskovan S., Wargo J.A., Ribas A. (2017). Primary, Adaptive, and Acquired Resistance to Cancer Immunotherapy. Cell.

[3] Del Poggetto E., Ho I.L., Balestrieri C., Yen E.Y., Zhang S., Citron F., Shah R., Corti D., Diaferia G.R., Li C.Y. (2021). Epithelial memory of inflammation limits tissue damage while promoting pancreatic tumorigenesis. Science.

[4] Hanahan D., Coussens L.M. (2012). Accessories to the crime: Functions of cells recruited to the tumor microenvironment. Cancer Cell.

[5] Hiam-Galvez K.J., Allen B.M., Spitzer M.H. (2021). Systemic immunity in cancer. Nat. Rev. Cancer.

[6] Leach D.R., Krummel M.F., Allison J.P. (1996). Enhancement of antitumor immunity by CTLA-4 blockade. Science.

[7] Iwai Y., Terawaki S., Honjo T. (2005). PD-1 blockade inhibits hematogenous spread of poorly immunogenic tumor cells by enhanced recruitment of effector T cells. Int. Immunol..

[8] Qin S., Xu L., Yi M., Yu S., Wu K., Luo S. (2019). Novel immune checkpoint targets: Moving beyond PD-1 and CTLA-4. Mol. Cancer.

[9] Robert C. (2020). A decade of immune-checkpoint inhibitors in cancer therapy. Nat. Commun..

[10] Robert C., Ribas A., Hamid O., Daud A., Wolchok J.D., Joshua A.M., Hwu W.J., Weber J.S., Gangadhar T.C., Joseph R.W. (2018). Durable Complete Response After Discontinuation of Pembrolizumab in Patients with Metastatic Melanoma. J. Clin. Oncol. Off. J. Am. Soc. Clin. Oncol..

[11] Larkin J., Lao C.D., Urba W.J., McDermott D.F., Horak C., Jiang J., Wolchok J.D. (2015). Efficacy and Safety of Nivolumab in Patients with BRAF V600 Mutant and BRAF Wild-Type Advanced Melanoma: A Pooled Analysis of 4 Clinical Trials. JAMA Oncol..

[12] Rosenberg S.A., Yang J.C., Sherry R.M., Kammula U.S., Hughes M.S., Phan G.Q., Citrin D.E., Restifo N.P., Robbins P.F., Wunderlich J.R. (2011). Durable complete responses in heavily pretreated patients with metastatic melanoma using T-cell transfer immunotherapy. Clin. Cancer Res. Off. J. Am. Assoc. Cancer Res..

[13] Kirtane K., Elmariah H., Chung C.H., Abate-Daga D. (2021). Adoptive cellular therapy in solid tumor malignancies: Review of the literature and challenges ahead. J. Immunother. Cancer.

[14] Yee C., Lizee G., Schueneman A.J. (2015). Endogenous T-Cell Therapy: Clinical Experience. Cancer J..

[15] Sadelain M., Brentjens R., Rivière I. (2013). The basic principles of chimeric antigen receptor design. Cancer Discov..

[16] June C.H. (2007). Adoptive T cell therapy for cancer in the clinic. J. Clin. Investig..

[17] Sterner R.C., Sterner R.M. (2021). CAR-T cell therapy: Current limitations and potential strategies. Blood Cancer J..

[18] Maude S.L., Frey N., Shaw P.A., Aplenc R., Barrett D.M., Bunin N.J., Chew A., Gonzalez V.E., Zheng Z., Lacey S.F. (2014). Chimeric antigen receptor T cells for sustained remissions in leukemia. N. Engl. J. Med..

[19] Met Ö., Jensen K.M., Chamberlain C.A., Donia M., Svane I.M. (2019). Principles of adoptive T cell therapy in cancer. Semin. Immunopathol..

[20] Yang J.C., Rosenberg S.A. (2016). Adoptive T-Cell Therapy for Cancer. Adv. Immunol..

[21] Sadelain M. (2016). Chimeric antigen receptors: Driving immunology towards synthetic biology. Curr. Opin. Immunol..

[22] Kankeu Fonkoua L.A., Sirpilla O., Sakemura R., Siegler E.L., Kenderian S.S. (2022). CAR T cell therapy and the tumor microenvironment: Current challenges and opportunities. Mol. Ther. Oncolytics.

[23] Kalbasi A., Siurala M., Su L.L., Tariveranmoshabad M., Picton L.K., Ravikumar P., Li P., Lin J.-X., Escuin-Ordinas H., Da T. (2022). Potentiating adoptive cell therapy using synthetic IL-9 receptors. Nature.

[24] Liu S., Galat V., Galat Y., Lee Y.K.A., Wainwright D., Wu J. (2021). NK cell-based cancer immunotherapy: From basic biology to clinical development. J. Hematol. Oncol..

[25] Yang L., Li A., Lei Q., Zhang Y. (2019). Tumor-intrinsic signaling pathways: Key roles in the regulation of the immunosuppressive tumor microenvironment. J. Hematol. Oncol..

[26] Liu C., Peng W., Xu C., Lou Y., Zhang M., Wargo J.A., Chen J.Q., Li H.S., Watowich S.S., Yang Y. (2013). BRAF inhibition increases tumor infiltration by T cells and enhances the antitumor activity of adoptive immunotherapy in mice. Clin. Cancer Res. Off. J. Am. Assoc. Cancer Res..

[27] Loi S., Dushyanthen S., Beavis P.A., Salgado R., Denkert C., Savas P., Combs S., Rimm D.L., Giltnane J.M., Estrada M.V. (2016). RAS/MAPK Activation Is Associated with Reduced Tumor-Infiltrating Lymphocytes in Triple-Negative Breast Cancer: Therapeutic Cooperation Between MEK and PD-1/PD-L1 Immune Checkpoint Inhibitors. Clin. Cancer Res. Off. J. Am. Assoc. Cancer Res..

[28] Hu-Lieskovan S., Mok S., Homet Moreno B., Tsoi J., Robert L., Goedert L., Pinheiro E.M., Koya R.C., Graeber T.G., Comin-Anduix B. (2015). Improved antitumor activity of immunotherapy with BRAF and MEK inhibitors in BRAF(V600E) melanoma. Sci. Transl. Med..

[29] Liu L., Mayes P.A., Eastman S., Shi H., Yadavilli S., Zhang T., Yang J., Seestaller-Wehr L., Zhang S.Y., Hopson C. (2015). The BRAF and MEK Inhibitors Dabrafenib and Trametinib: Effects on Immune Function and in Combination with Immunomodulatory Antibodies Targeting PD-1, PD-L1, and CTLA-4. Clin. Cancer Res. Off. J. Am. Assoc. Cancer Res..

[30] Peng W., Chen J.Q., Liu C., Malu S., Creasy C., Tetzlaff M.T., Xu C., McKenzie J.A., Zhang C., Liang X. (2016). Loss of PTEN Promotes Resistance to T Cell-Mediated Immunotherapy. Cancer Discov..

[31] Zhao J., Chen A.X., Gartrell R.D., Silverman A.M., Aparicio L., Chu T., Bordbar D., Shan D., Samanamud J., Mahajan A. (2019). Immune and genomic correlates of response to anti-PD-1 immunotherapy in glioblastoma. Nat. Med..

[32] Spranger S., Bao R., Gajewski T.F. (2015). Melanoma-intrinsic β-catenin signalling prevents anti-tumour immunity. Nature.

[33] Luke J.J., Bao R., Sweis R.F., Spranger S., Gajewski T.F. (2019). WNT/β-catenin Pathway Activation Correlates with Immune Exclusion across Human Cancers. Clin. Cancer Res. Off. J. Am. Assoc. Cancer Res..

[34] Darnell J.E., Kerr I.M., Stark G.R. (1994). Jak-STAT pathways and transcriptional activation in response to IFNs and other extracellular signaling proteins. Science.

[35] Platanias L.C. (2005). Mechanisms of type-I- and type-II-interferon-mediated signalling. Nat. Rev. Immunol..

[36] Dunn G.P., Bruce A.T., Sheehan K.C., Shankaran V., Uppaluri R., Bui J.D., Diamond M.S., Koebel C.M., Arthur C., White J.M. (2005). A critical function for type I interferons in cancer immunoediting. Nat. Immunol..

[37] Benci J.L., Xu B., Qiu Y., Wu T.J., Dada H., Twyman-Saint Victor C., Cucolo L., Lee D.S.M., Pauken K.E., Huang A.C. (2016). Tumor Interferon Signaling Regulates a Multigenic Resistance Program to Immune Checkpoint Blockade. Cell.

[38] Gao J., Shi L.Z., Zhao H., Chen J., Xiong L., He Q., Chen T., Roszik J., Bernatchez C., Woodman S.E. (2016). Loss of IFN-γ Pathway Genes in Tumor Cells as a Mechanism of Resistance to Anti-CTLA-4 Therapy. Cell.

[39] Shin D.S., Zaretsky J.M., Escuin-Ordinas H., Garcia-Diaz A., Hu-Lieskovan S., Kalbasi A., Grasso C.S., Hugo W., Sandoval S., Torrejon D.Y. (2017). Primary Resistance to PD-1 Blockade Mediated by JAK1/2 Mutations. Cancer Discov..

[40] Maeurer M.J., Gollin S.M., Storkus W.J., Swaney W., Karbach J., Martin D., Castelli C., Salter R., Knuth A., Lotze M.T. (1996). Tumor escape from immune recognition: Loss of HLA-A2 melanoma cell surface expression is associated with a complex rearrangement of the short arm of chromosome 6. Clin. Cancer Res. Off. J. Am. Assoc. Cancer Res..

[41] Hazini A., Fisher K., Seymour L. (2021). Deregulation of HLA-I in cancer and its central importance for immunotherapy. J. Immunother. Cancer.

[42] Taylor B.C., Balko J.M. (2022). Mechanisms of MHC-I Downregulation and Role in Immunotherapy Response. Front. Immunol..

[43] Yoshihama S., Roszik J., Downs I., Meissner T.B., Vijayan S., Chapuy B., Sidiq T., Shipp M.A., Lizee G.A., Kobayashi K.S. (2016). NLRC5/MHC class I transactivator is a target for immune evasion in cancer. Proc. Natl. Acad. Sci. USA.

[44] Cornel A.M., Mimpen I.L., Nierkens S. (2020). MHC Class I Downregulation in Cancer: Underlying Mechanisms and Potential Targets for Cancer Immunotherapy. Cancers.

[45] Garrido F. (2019). MHC/HLA Class I Loss in Cancer Cells. Adv. Exp. Med. Biol..

[46] Tanaka K., Tsuchikawa T., Miyamoto M., Maki T., Ichinokawa M., Kubota K.C., Shichinohe T., Hirano S., Ferrone S., Dosaka-Akita H. (2012). Down-regulation of Human Leukocyte Antigen class I heavy chain in tumors is associated with a poor prognosis in advanced esophageal cancer patients. Int. J. Oncol..

[47] Kim H.J., Bae S.C. (2011). Histone deacetylase inhibitors: Molecular mechanisms of action and clinical trials as anti-cancer drugs. Am. J. Transl. Res..

[48] Roulois D., Yau H.L., De Carvalho D.D. (2016). Pharmacological DNA demethylation: Implications for cancer immunotherapy. Oncoimmunology.

[49] Kirkin A.F., Dzhandzhugazyan K.N., Guldberg P., Fang J.J., Andersen R.S., Dahl C., Mortensen J., Lundby T., Wagner A., Law I. (2018). Adoptive cancer immunotherapy using DNA-demethylated T helper cells as antigen-presenting cells. Nat. Commun..

[50] Jung H., Kim H.S., Kim J.Y., Sun J.M., Ahn J.S., Ahn M.J., Park K., Esteller M., Lee S.H., Choi J.K. (2019). DNA methylation loss promotes immune evasion of tumours with high mutation and copy number load. Nat. Commun..

[51] Rosborough B.R., Castellaneta A., Natarajan S., Thomson A.W., Turnquist H.R. (2012). Histone deacetylase inhibition facilitates GM-CSF-mediated expansion of myeloid-derived suppressor cells in vitro and in vivo. J. Leukoc. Biol..

[52] Wang H.F., Ning F., Liu Z.C., Wu L., Li Z.Q., Qi Y.F., Zhang G., Wang H.S., Cai S.H., Du J. (2017). Histone deacetylase inhibitors deplete myeloid-derived suppressor cells induced by 4T1 mammary tumors in vivo and in vitro. Cancer Immunol. Immunother. CII.

[53] Ohue Y., Nishikawa H. (2019). Regulatory T (Treg) cells in cancer: Can Treg cells be a new therapeutic target?. Cancer Sci..

[54] Sundstedt A., O’Neill E.J., Nicolson K.S., Wraith D.C. (2003). Role for IL-10 in suppression mediated by peptide-induced regulatory T cells in vivo. J. Immunol..

[55] Sakaguchi S., Yamaguchi T., Nomura T., Ono M. (2008). Regulatory T cells and immune tolerance. Cell.

[56] Oida T., Zhang X., Goto M., Hachimura S., Totsuka M., Kaminogawa S., Weiner H.L. (2003). CD4+CD25- T cells that express latency-associated peptide on the surface suppress CD4+CD45RBhigh-induced colitis by a TGF-beta-dependent mechanism. J. Immunol..

[57] Nishikawa H., Koyama S. (2021). Mechanisms of regulatory T cell infiltration in tumors: Implications for innovative immune precision therapies. J. Immunother. Cancer.

[58] Quezada S.A., Peggs K.S., Curran M.A., Allison J.P. (2006). CTLA4 blockade and GM-CSF combination immunotherapy alters the intratumor balance of effector and regulatory T cells. J. Clin. Investig..

[59] Simpson T.R., Li F., Montalvo-Ortiz W., Sepulveda M.A., Bergerhoff K., Arce F., Roddie C., Henry J.Y., Yagita H., Wolchok J.D. (2013). Fc-dependent depletion of tumor-infiltrating regulatory T cells co-defines the efficacy of anti-CTLA-4 therapy against melanoma. J. Exp. Med..

[60] Arce Vargas F., Furness A.J.S., Litchfield K., Joshi K., Rosenthal R., Ghorani E., Solomon I., Lesko M.H., Ruef N., Roddie C. (2018). Fc Effector Function Contributes to the Activity of Human Anti-CTLA-4 Antibodies. Cancer Cell.

[61] Hamid O., Schmidt H., Nissan A., Ridolfi L., Aamdal S., Hansson J., Guida M., Hyams D.M., Gómez H., Bastholt L. (2011). A prospective phase II trial exploring the association between tumor microenvironment biomarkers and clinical activity of ipilimumab in advanced melanoma. J. Transl. Med..

[62] Sharma A., Subudhi S.K., Blando J., Scutti J., Vence L., Wargo J., Allison J.P., Ribas A., Sharma P. (2019). Anti-CTLA-4 Immunotherapy Does Not Deplete FOXP3(+) Regulatory T Cells (Tregs) in Human Cancers. Clin. Cancer Res. Off. J. Am. Assoc. Cancer Res..

[63] Veglia F., Sanseviero E., Gabrilovich D.I. (2021). Myeloid-derived suppressor cells in the era of increasing myeloid cell diversity. Nat. Rev. Immunol..

[64] Wesolowski R., Markowitz J., Carson W.E. (2013). Myeloid derived suppressor cells—A new therapeutic target in the treatment of cancer. J. Immunother. Cancer.

[65] Yang L., DeBusk L.M., Fukuda K., Fingleton B., Green-Jarvis B., Shyr Y., Matrisian L.M., Carbone D.P., Lin P.C. (2004). Expansion of myeloid immune suppressor Gr+CD11b+ cells in tumor-bearing host directly promotes tumor angiogenesis. Cancer Cell.

[66] Yang L., Huang J., Ren X., Gorska A.E., Chytil A., Aakre M., Carbone D.P., Matrisian L.M., Richmond A., Lin P.C. (2008). Abrogation of TGF beta signaling in mammary carcinomas recruits Gr-1+CD11b+ myeloid cells that promote metastasis. Cancer Cell.

[67] Li K., Shi H., Zhang B., Ou X., Ma Q., Chen Y., Shu P., Li D., Wang Y. (2021). Myeloid-derived suppressor cells as immunosuppressive regulators and therapeutic targets in cancer. Signal Transduct. Target. Ther..

[68] Solito S., Falisi E., Diaz-Montero C.M., Doni A., Pinton L., Rosato A., Francescato S., Basso G., Zanovello P., Onicescu G. (2011). A human promyelocytic-like population is responsible for the immune suppression mediated by myeloid-derived suppressor cells. Blood.

[69] Si Y., Merz S.F., Jansen P., Wang B., Bruderek K., Altenhoff P., Mattheis S., Lang S., Gunzer M., Klode J. (2019). Multidimensional imaging provides evidence for down-regulation of T cell effector function by MDSC in human cancer tissue. Sci. Immunol..

[70] Kodumudi K.N., Weber A., Sarnaik A.A., Pilon-Thomas S. (2012). Blockade of myeloid-derived suppressor cells after induction of lymphopenia improves adoptive T cell therapy in a murine model of melanoma. J. Immunol..

[71] Meyer C., Cagnon L., Costa-Nunes C.M., Baumgaertner P., Montandon N., Leyvraz L., Michielin O., Romano E., Speiser D.E. (2014). Frequencies of circulating MDSC correlate with clinical outcome of melanoma patients treated with ipilimumab. Cancer Immunol. Immunother. CII.

[72] Kaneda M.M., Messer K.S., Ralainirina N., Li H., Leem C.J., Gorjestani S., Woo G., Nguyen A.V., Figueiredo C.C., Foubert P. (2016). PI3Kγ is a molecular switch that controls immune suppression. Nature.

[73] De Henau O., Rausch M., Winkler D., Campesato L.F., Liu C., Cymerman D.H., Budhu S., Ghosh A., Pink M., Tchaicha J. (2016). Overcoming resistance to checkpoint blockade therapy by targeting PI3Kγ in myeloid cells. Nature.

[74] Pathria P., Louis T.L., Varner J.A. (2019). Targeting Tumor-Associated Macrophages in Cancer. Trends Immunol..

[75] Xiang X., Wang J., Lu D., Xu X. (2021). Targeting tumor-associated macrophages to synergize tumor immunotherapy. Signal Transduct. Target. Ther..

[76] Lin Y., Xu J., Lan H. (2019). Tumor-associated macrophages in tumor metastasis: Biological roles and clinical therapeutic applications. J. Hematol. Oncol..

[77] Neubert N.J., Schmittnaegel M., Bordry N., Nassiri S., Wald N., Martignier C., Tillé L., Homicsko K., Damsky W., Maby-El Hajjami H. (2018). T cell-induced CSF1 promotes melanoma resistance to PD1 blockade. Sci. Transl. Med..

[78] Mok S., Koya R.C., Tsui C., Xu J., Robert L., Wu L., Graeber T., West B.L., Bollag G., Ribas A. (2014). Inhibition of CSF-1 receptor improves the antitumor efficacy of adoptive cell transfer immunotherapy. Cancer Res..

[79] Zhu Y., Yang J., Xu D., Gao X.M., Zhang Z., Hsu J.L., Li C.W., Lim S.O., Sheng Y.Y., Zhang Y. (2019). Disruption of tumour-associated macrophage trafficking by the osteopontin-induced colony-stimulating factor-1 signalling sensitises hepatocellular carcinoma to anti-PD-L1 blockade. Gut.

[80] Zhu Y., Knolhoff B.L., Meyer M.A., Nywening T.M., West B.L., Luo J., Wang-Gillam A., Goedegebuure S.P., Linehan D.C., DeNardo D.G. (2014). CSF1/CSF1R blockade reprograms tumor-infiltrating macrophages and improves response to T-cell checkpoint immunotherapy in pancreatic cancer models. Cancer Res..

[81] Hu W., Li X., Zhang C., Yang Y., Jiang J., Wu C. (2016). Tumor-associated macrophages in cancers. Clin. Transl. Oncol..

[82] Jung K.Y., Cho S.W., Kim Y.A., Kim D., Oh B.C., Park D.J., Park Y.J. (2015). Cancers with Higher Density of Tumor-Associated Macrophages Were Associated with Poor Survival Rates. J. Pathol. Transl. Med..

[83] Fritz J.M., Tennis M.A., Orlicky D.J., Lin H., Ju C., Redente E.F., Choo K.S., Staab T.A., Bouchard R.J., Merrick D.T. (2014). Depletion of tumor-associated macrophages slows the growth of chemically induced mouse lung adenocarcinomas. Front. Immunol..

[84] Wu X., Schulte B.C., Zhou Y., Haribhai D., Mackinnon A.C., Plaza J.A., Williams C.B., Hwang S.T. (2014). Depletion of M2-like tumor-associated macrophages delays cutaneous T-cell lymphoma development in vivo. J. Investig. Dermatol..

[85] Ries C.H., Cannarile M.A., Hoves S., Benz J., Wartha K., Runza V., Rey-Giraud F., Pradel L.P., Feuerhake F., Klaman I. (2014). Targeting tumor-associated macrophages with anti-CSF-1R antibody reveals a strategy for cancer therapy. Cancer Cell.

[86] Luo Y., Zhou H., Krueger J., Kaplan C., Lee S.H., Dolman C., Markowitz D., Wu W., Liu C., Reisfeld R.A. (2006). Targeting tumor-associated macrophages as a novel strategy against breast cancer. J. Clin. Investig..

[87] Ruffell B., Chang-Strachan D., Chan V., Rosenbusch A., Ho C.M., Pryer N., Daniel D., Hwang E.S., Rugo H.S., Coussens L.M. (2014). Macrophage IL-10 blocks CD8+ T cell-dependent responses to chemotherapy by suppressing IL-12 expression in intratumoral dendritic cells. Cancer Cell.

[88] Shaked Y. (2019). The pro-tumorigenic host response to cancer therapies. Nat. Rev. Cancer.

[89] Gajewski T.F., Schreiber H., Fu Y.X. (2013). Innate and adaptive immune cells in the tumor microenvironment. Nat. Immunol..

[90] Gray-Owen S.D., Blumberg R.S. (2006). CEACAM1: Contact-dependent control of immunity. Nat. Rev. Immunol..

[91] Seoane J., Gomis R.R. (2017). TGF-β Family Signaling in Tumor Suppression and Cancer Progression. Cold Spring Harb. Perspect. Biol..

[92] Massagué J. (2008). TGFbeta in Cancer. Cell.

[93] Hanks B.A., Holtzhausen A., Evans K., Heid M., Blobe G.C. (2014). Combinatorial TGF-β signaling blockade and anti-CTLA-4 antibody immunotherapy in a murine BRAFV600E-PTEN-/- transgenic model of melanoma. J. Clin. Oncol..

[94] Vanpouille-Box C., Diamond J.M., Pilones K.A., Zavadil J., Babb J.S., Formenti S.C., Barcellos-Hoff M.H., Demaria S. (2015). TGFβ Is a Master Regulator of Radiation Therapy-Induced Antitumor Immunity. Cancer Res..

[95] Kohli K., Pillarisetty V.G., Kim T.S. (2022). Key chemokines direct migration of immune cells in solid tumors. Cancer Gene Ther..

[96] Highfill S.L., Cui Y., Giles A.J., Smith J.P., Zhang H., Morse E., Kaplan R.N., Mackall C.L. (2014). Disruption of CXCR2-mediated MDSC tumor trafficking enhances anti-PD1 efficacy. Sci. Transl. Med..

[97] Yang H., Zhang Q., Xu M., Wang L., Chen X., Feng Y., Li Y., Zhang X., Cui W., Jia X. (2020). CCL2-CCR2 axis recruits tumor associated macrophages to induce immune evasion through PD-1 signaling in esophageal carcinogenesis. Mol. Cancer.

[98] Sugiyama D., Nishikawa H., Maeda Y., Nishioka M., Tanemura A., Katayama I., Ezoe S., Kanakura Y., Sato E., Fukumori Y. (2013). Anti-CCR4 mAb selectively depletes effector-type FoxP3+CD4+ regulatory T cells, evoking antitumor immune responses in humans. Proc. Natl. Acad. Sci. USA.

[99] Chang D.K., Sui J., Geng S., Muvaffak A., Bai M., Fuhlbrigge R.C., Lo A., Yammanuru A., Hubbard L., Sheehan J. (2012). Humanization of an anti-CCR4 antibody that kills cutaneous T-cell lymphoma cells and abrogates suppression by T-regulatory cells. Mol. Cancer Ther..

[100] Bockorny B., Semenisty V., Macarulla T., Borazanci E., Wolpin B.M., Stemmer S.M., Golan T., Geva R., Borad M.J., Pedersen K.S. (2020). BL-8040, a CXCR4 antagonist, in combination with pembrolizumab and chemotherapy for pancreatic cancer: The COMBAT trial. Nat. Med..

[101] Sahai E., Astsaturov I., Cukierman E., DeNardo D.G., Egeblad M., Evans R.M., Fearon D., Greten F.R., Hingorani S.R., Hunter T. (2020). A framework for advancing our understanding of cancer-associated fibroblasts. Nat. Rev. Cancer.

[102] Servais C., Erez N. (2013). From sentinel cells to inflammatory culprits: Cancer-associated fibroblasts in tumour-related inflammation. J. Pathol..

[103] Raz Y., Cohen N., Shani O., Bell R.E., Novitskiy S.V., Abramovitz L., Levy C., Milyavsky M., Leider-Trejo L., Moses H.L. (2018). Bone marrow–derived fibroblasts are a functionally distinct stromal cell population in breast cancer. J. Exp. Med..

[104] Klein S.L., Flanagan K.L. (2016). Sex differences in immune responses. Nat. Rev. Immunol..

[105] Conforti F., Pala L., Bagnardi V., De Pas T., Martinetti M., Viale G., Gelber R.D., Goldhirsch A. (2018). Cancer immunotherapy efficacy and patients’ sex: A systematic review and meta-analysis. Lancet Oncol..

[106] Ford K., Hanley C.J., Mellone M., Szyndralewiez C., Heitz F., Wiesel P., Wood O., Machado M., Lopez M.A., Ganesan A.P. (2020). NOX4 Inhibition Potentiates Immunotherapy by Overcoming Cancer-Associated Fibroblast-Mediated CD8 T-cell Exclusion from Tumors. Cancer Res..

[107] Nishijima T.F., Muss H.B., Shachar S.S., Moschos S.J. (2016). Comparison of efficacy of immune checkpoint inhibitors (ICIs) between younger and older patients: A systematic review and meta-analysis. Cancer Treat. Rev..

[108] Murphy W.J., Longo D.L. (2019). The Surprisingly Positive Association Between Obesity and Cancer Immunotherapy Efficacy. JAMA.

[109] Jacobi O., Landman Y., Reinhorn D., Icht O., Sternschuss M., Rotem O., Finkel I., Allen A.M., Dudnik E., Goldstein D.A. (2021). The Relationship of Diabetes Mellitus to Efficacy of Immune Checkpoint Inhibitors in Patients with Advanced Non-Small Cell Lung Cancer. Oncology.

[110] Cani P.D. (2018). Human gut microbiome: Hopes, threats and promises. Gut.

[111] Zhou C.B., Zhou Y.L., Fang J.Y. (2021). Gut Microbiota in Cancer Immune Response and Immunotherapy. Trends Cancer.

[112] Vétizou M., Pitt J.M., Daillère R., Lepage P., Waldschmitt N., Flament C., Rusakiewicz S., Routy B., Roberti M.P., Duong C.P. (2015). Anticancer immunotherapy by CTLA-4 blockade relies on the gut microbiota. Science.

[113] Sivan A., Corrales L., Hubert N., Williams J.B., Aquino-Michaels K., Earley Z.M., Benyamin F.W., Lei Y.M., Jabri B., Alegre M.L. (2015). Commensal Bifidobacterium promotes antitumor immunity and facilitates anti-PD-L1 efficacy. Science.

[114] Gopalakrishnan V., Spencer C.N., Nezi L., Reuben A., Andrews M.C., Karpinets T.V., Prieto P.A., Vicente D., Hoffman K., Wei S.C. (2018). Gut microbiome modulates response to anti-PD-1 immunotherapy in melanoma patients. Science.

[115] Routy B., Le Chatelier E., Derosa L., Duong C.P.M., Alou M.T., Daillère R., Fluckiger A., Messaoudene M., Rauber C., Roberti M.P. (2018). Gut microbiome influences efficacy of PD-1-based immunotherapy against epithelial tumors. Science.

[116] Matson V., Fessler J., Bao R., Chongsuwat T., Zha Y., Alegre M.L., Luke J.J., Gajewski T.F. (2018). The commensal microbiome is associated with anti-PD-1 efficacy in metastatic melanoma patients. Science.

[117] Griffin Matthew E., Espinosa J., Becker Jessica L., Luo J.-D., Carroll Thomas S., Jha Jyoti K., Fanger Gary R., Hang Howard C. (2021). Enterococcus peptidoglycan remodeling promotes checkpoint inhibitor cancer immunotherapy. Science.

[118] Tray N., Weber J.S., Adams S. (2018). Predictive Biomarkers for Checkpoint Immunotherapy: Current Status and Challenges for Clinical Application. Cancer Immunol. Res..

[119] Roszik J., Haydu L.E., Hess K.R., Oba J., Joon A.Y., Siroy A.E., Karpinets T.V., Stingo F.C., Baladandayuthapani V., Tetzlaff M.T. (2016). Novel algorithmic approach predicts tumor mutation load and correlates with immunotherapy clinical outcomes using a defined gene mutation set. BMC Med..

[120] Chen P.L., Roh W., Reuben A., Cooper Z.A., Spencer C.N., Prieto P.A., Miller J.P., Bassett R.L., Gopalakrishnan V., Wani K. (2016). Analysis of Immune Signatures in Longitudinal Tumor Samples Yields Insight into Biomarkers of Response and Mechanisms of Resistance to Immune Checkpoint Blockade. Cancer Discov..

[121] Liu Y.T., Sun Z.J. (2021). Turning cold tumors into hot tumors by improving T-cell infiltration. Theranostics.

[122] Corrales L., Glickman L.H., McWhirter S.M., Kanne D.B., Sivick K.E., Katibah G.E., Woo S.R., Lemmens E., Banda T., Leong J.J. (2015). Direct Activation of STING in the Tumor Microenvironment Leads to Potent and Systemic Tumor Regression and Immunity. Cell Rep..

[123] Holmgaard R.B., Zamarin D., Munn D.H., Wolchok J.D., Allison J.P. (2013). Indoleamine 2,3-dioxygenase is a critical resistance mechanism in antitumor T cell immunotherapy targeting CTLA-4. J. Exp. Med..

[124] Edenfield W.J., Chung K., O’Rourke M., Cull E., Martin J., Bowers H., Smith W., Gluck W.L. (2021). A Phase II Study of Durvalumab in Combination with Tremelimumab in Patients with Rare Cancers. Oncologist.

[125] Olson D.J., Eroglu Z., Brockstein B., Poklepovic A.S., Bajaj M., Babu S., Hallmeyer S., Velasco M., Lutzky J., Higgs E. (2021). Pembrolizumab Plus Ipilimumab Following Anti-PD-1/L1 Failure in Melanoma. J. Clin. Oncol. Off. J. Am. Soc. Clin. Oncol..

[126] Friedman C.F., Spencer C., Cabanski C.R., Panageas K.S., Wells D.K., Ribas A., Tawbi H., Tsai K., Postow M., Shoushtari A. (2022). Ipilimumab alone or in combination with nivolumab in patients with advanced melanoma who have progressed or relapsed on PD-1 blockade: Clinical outcomes and translational biomarker analyses. J. Immunother. Cancer.

[127] Gibney G.T., Hamid O., Lutzky J., Olszanski A.J., Mitchell T.C., Gajewski T.F., Chmielowski B., Hanks B.A., Zhao Y., Newton R.C. (2019). Phase 1/2 study of epacadostat in combination with ipilimumab in patients with unresectable or metastatic melanoma. J. Immunother. Cancer.

[128] Zakharia Y., McWilliams R.R., Rixe O., Drabick J., Shaheen M.F., Grossmann K.F., Kolhe R., Pacholczyk R., Sadek R., Tennant L.L. (2021). Phase II trial of the IDO pathway inhibitor indoximod plus pembrolizumab for the treatment of patients with advanced melanoma. J. Immunother. Cancer.

[129] Hawkes E.A., Phillips T., Budde L.E., Santoro A., Saba N.S., Roncolato F., Gregory G.P., Verhoef G., Offner F., Quero C. (2021). Avelumab in Combination Regimens for Relapsed/Refractory DLBCL: Results from the Phase Ib JAVELIN DLBCL Study. Target. Oncol..

[130] Burris H.A., Callahan M.K., Tolcher A.W., Kummar S., Falchook G.S., Pachynski R.K., Tykodi S.S., Gibney G.T., Seiwert T.Y., Gainor J.F. (2017). Phase 1 safety of ICOS agonist antibody JTX-2011 alone and with nivolumab (nivo) in advanced solid tumors; predicted vs observed pharmacokinetics (PK) in ICONIC. J. Clin. Oncol..

[131] Felip E., Lim F.L., Johnson M., O’Brien M., Barlesi F., Mazieres J., Solomon B., Moreno V., Boni V., Swalduz A. (2020). 1315P Phase Ib/II open-label, randomised evaluation of atezolizumab (atezo) + CPI-444 vs docetaxel as second/third-line therapy in MORPHEUS-NSCLC (non-small cell lung cancer). Ann. Oncol..

[132] Sanborn R.E., Pishvaian M.J., Callahan M.K., Weise A., Sikic B.I., Rahma O., Cho D.C., Rizvi N.A., Sznol M., Lutzky J. (2022). Safety, tolerability and efficacy of agonist anti-CD27 antibody (varlilumab) administered in combination with anti-PD-1 (nivolumab) in advanced solid tumors. J. Immunother. Cancer.

[133] Diab A., Tannir N.M., Bentebibel S.E., Hwu P., Papadimitrakopoulou V., Haymaker C., Kluger H.M., Gettinger S.N., Sznol M., Tykodi S.S. (2020). Bempegaldesleukin (NKTR-214) plus Nivolumab in Patients with Advanced Solid Tumors: Phase I Dose-Escalation Study of Safety, Efficacy, and Immune Activation (PIVOT-02). Cancer Discov..

[134] O’Day S., Borges V., Chmielowski B., Rao R., Abu-Khalaf M., Stopeck A., Lowe J., Mattson P., Breuer K., Gargano M. (2019). Abstract P2-09-08: Imprime PGG, a novel innate immune modulator, combined with pembrolizumab in a phase 2 multicenter, open label study in chemotherapy-resistant metastatic triple negative breast cancer (TNBC). Cancer Res..

[135] Tolcher A.W., Sznol M., Hu-Lieskovan S., Papadopoulos K.P., Patnaik A., Rasco D.W., Di Gravio D., Huang B., Gambhire D., Chen Y. (2017). Phase Ib Study of Utomilumab (PF-05082566), a 4-1BB/CD137 Agonist, in Combination with Pembrolizumab (MK-3475) in Patients with Advanced Solid Tumors. Clin. Cancer Res. Off. J. Am. Assoc. Cancer Res..

[136] Cassier P.A., Garin G., Eberst L., Delord J.-P., Chabaud S., Terret C., Montane L., Bidaux A.-S., Laurent S., Jaubert L. (2019). MEDIPLEX: A phase 1 study of durvalumab (D) combined with pexidartinib (P) in patients (pts) with advanced pancreatic ductal adenocarcinoma (PDAC) and colorectal cancer (CRC). J. Clin. Oncol..

[137] Lin C.-C., Gil-Martin M., Bauer T.M., Naing A., Lim D.W.-T., Sarantopoulos J., Geva R., Ando Y., Fan L., Choudhury S. (2020). Abstract CT171: Phase I study of BLZ945 alone and with spartalizumab (PDR001) in patients (pts) with advanced solid tumors. Cancer Res..

[138] Chesney J., Puzanov I., Collichio F., Singh P., Milhem M.M., Glaspy J., Hamid O., Ross M., Friedlander P., Garbe C. (2018). Randomized, Open-Label Phase II Study Evaluating the Efficacy and Safety of Talimogene Laherparepvec in Combination with Ipilimumab Versus Ipilimumab Alone in Patients with Advanced, Unresectable Melanoma. J. Clin. Oncol. Off. J. Am. Soc. Clin. Oncol..

[139] Wong D.J.L., Panwar A., Rosenberg A., Karivedu V., Laux D.E., Zandberg D.P., Bobilev D., Zhao L., Wooldridge J., Krieg A.M. (2021). CMP-001-007: Open-label, phase 2 study of intratumoral CMP-001 + pembrolizumab in patients with recurrent or metastatic head and neck squamous cell carcinoma. J. Clin. Oncol..

[140] Ribas A., Medina T., Kummar S., Amin A., Kalbasi A., Drabick J.J., Barve M., Daniels G.A., Wong D.J., Schmidt E.V. (2018). SD-101 in Combination with Pembrolizumab in Advanced Melanoma: Results of a Phase Ib, Multicenter Study. Cancer Discov..

[141] Zamarin D., Walderich S., Holland A., Zhou Q., Iasonos A.E., Torrisi J.M., Merghoub T., Chesebrough L.F., McDonnell A.S., Gallagher J.M. (2020). Safety, immunogenicity, and clinical efficacy of durvalumab in combination with folate receptor alpha vaccine TPIV200 in patients with advanced ovarian cancer: A phase II trial. J. Immunother. Cancer.

[142] Tsujikawa T., Crocenzi T., Durham J.N., Sugar E.A., Wu A.A., Onners B., Nauroth J.M., Anders R.A., Fertig E.J., Laheru D.A. (2020). Evaluation of Cyclophosphamide/GVAX Pancreas Followed by Listeria-Mesothelin (CRS-207) with or without Nivolumab in Patients with Pancreatic Cancer. Clin. Cancer Res. Off. J. Am. Assoc. Cancer Res..

[143] Rajan A., Gray J.E., Devarakonda S., Gurtler J., Birhiray R., Paschold E., Dasgupta A., Heery C., Pico-Navarro C., Piechatzek M. (2019). 1207P—Phase I trial of CV301 in combination with anti-PD-1 therapy in non-squamous NSCLC. Ann. Oncol..

[144] Xu R.-h., Mai H.-Q., Chen Q.-Y., Chen D., Hu C., Yang K., Wen J., Li J.-G., Shi Y., Jin F. (2021). JUPITER-02: Randomized, double-blind, phase III study of toripalimab or placebo plus gemcitabine and cisplatin as first-line treatment for recurrent or metastatic nasopharyngeal carcinoma (NPC). J. Clin. Oncol..

[145] Locke F.L., Westin J.R., Miklos D.B., Herrara A.F., Jacobson C.A., Lee J., Rossi J.M., Bot A., Xue A., Navale L. (2017). Phase 1 Results from ZUMA-6: Axicabtagene Ciloleucel (axi-cel; KTE-C19) in Combination with Atezolizumab for the Treatment of Patients with Refractory Diffuse Large B Cell Lymphoma (DLBCL). Blood.

[146] Chesney J.A., Lutzky J., Thomas S.S., Nieva J.J., Munoz Couselo E., Martin-Liberal J., Rodriguez-Moreno J.F., Cacovean A., Li H., Fardis M. (2019). A phase II study of autologous tumor infiltrating lymphocytes (TIL, LN-144/LN-145) in patients with solid tumors. J. Clin. Oncol..

[147] Rini B.I., Powles T., Atkins M.B., Escudier B., McDermott D.F., Suarez C., Bracarda S., Stadler W.M., Donskov F., Lee J.L. (2019). Atezolizumab plus bevacizumab versus sunitinib in patients with previously untreated metastatic renal cell carcinoma (IMmotion151): A multicentre, open-label, phase 3, randomised controlled trial. Lancet.

[148] Gutzmer R., Stroyakovskiy D., Gogas H., Robert C., Lewis K., Protsenko S., Pereira R.P., Eigentler T., Rutkowski P., Demidov L. (2020). Atezolizumab, vemurafenib, and cobimetinib as first-line treatment for unresectable advanced BRAF(V600) mutation-positive melanoma (IMspire150): Primary analysis of the randomised, double-blind, placebo-controlled, phase 3 trial. Lancet.

[149] Ribas A., Algazi A., Ascierto P.A., Butler M.O., Chandra S., Gordon M., Hernandez-Aya L., Lawrence D., Lutzky J., Miller W.H. (2020). PD-L1 blockade in combination with inhibition of MAPK oncogenic signaling in patients with advanced melanoma. Nat. Commun..

[150] Hodi F.S., Lawrence D., Lezcano C., Wu X., Zhou J., Sasada T., Zeng W., Giobbie-Hurder A., Atkins M.B., Ibrahim N. (2014). Bevacizumab plus ipilimumab in patients with metastatic melanoma. Cancer Immunol. Res..

[151] Hassel J.C., Lee S.B., Meiss F., Meier F., Dimitrakopoulou-Strauss A., Jäger D., Enk A.H. (2016). Vemurafenib and ipilimumab: A promising combination? Results of a case series. Oncoimmunology.

[152] Amin A., Plimack E.R., Ernstoff M.S., Lewis L.D., Bauer T.M., McDermott D.F., Carducci M., Kollmannsberger C., Rini B.I., Heng D.Y.C. (2018). Safety and efficacy of nivolumab in combination with sunitinib or pazopanib in advanced or metastatic renal cell carcinoma: The CheckMate 016 study. J. Immunother. Cancer.

[153] Ascierto P.A., Ferrucci P.F., Fisher R., Del Vecchio M., Atkinson V., Schmidt H., Schachter J., Queirolo P., Long G.V., Di Giacomo A.M. (2019). Dabrafenib, trametinib and pembrolizumab or placebo in BRAF-mutant melanoma. Nat. Med..

[154] Usmani S.Z., Schjesvold F., Oriol A., Karlin L., Cavo M., Rifkin R.M., Yimer H.A., LeBlanc R., Takezako N., McCroskey R.D. (2019). Pembrolizumab plus lenalidomide and dexamethasone for patients with treatment-naive multiple myeloma (KEYNOTE-185): A randomised, open-label, phase 3 trial. Lancet Haematol..

[155] Kelly K., Daly M.E., Mirhadi A., Lara F., Garcia L.A., Chen S., Eastham D., Wiegner E.A., Riess J.W., Schalper K.A. (2020). Atezolizumab plus stereotactic ablative therapy for medically inoperable patients with early-stage non-small cell lung cancer. J. Clin. Oncol..

[156] Theelen W., Peulen H.M.U., Lalezari F., van der Noort V., de Vries J.F., Aerts J., Dumoulin D.W., Bahce I., Niemeijer A.N., de Langen A.J. (2019). Effect of Pembrolizumab After Stereotactic Body Radiotherapy vs Pembrolizumab Alone on Tumor Response in Patients with Advanced Non-Small Cell Lung Cancer: Results of the PEMBRO-RT Phase 2 Randomized Clinical Trial. JAMA Oncol..

[157] Maity A., Mick R., Huang A.C., George S.M., Farwell M.D., Lukens J.N., Berman A.T., Mitchell T.C., Bauml J., Schuchter L.M. (2018). A phase I trial of pembrolizumab with hypofractionated radiotherapy in patients with metastatic solid tumours. Br. J. Cancer.

[158] Foster C.C., Fleming G.F., Karrison T.G., Liao C.Y., Desai A.V., Moroney J.W., Ratain M.J., Nanda R., Polite B.N., Hahn O.M. (2021). Phase I Study of Stereotactic Body Radiotherapy plus Nivolumab and Urelumab or Cabiralizumab in Advanced Solid Tumors. Clin. Cancer Res. Off. J. Am. Assoc. Cancer Res..

[159] Jotte R., Cappuzzo F., Vynnychenko I., Stroyakovskiy D., Rodríguez-Abreu D., Hussein M., Soo R., Conter H.J., Kozuki T., Huang K.C. (2020). Atezolizumab in Combination with Carboplatin and Nab-Paclitaxel in Advanced Squamous NSCLC (IMpower131): Results from a Randomized Phase III Trial. J. Thorac. Oncol. Off. Publ. Int. Assoc. Study Lung Cancer.

[160] Cortés J., André F., Gonçalves A., Kümmel S., Martín M., Schmid P., Schuetz F., Swain S.M., Easton V., Pollex E. (2019). IMpassion132 Phase III trial: Atezolizumab and chemotherapy in early relapsing metastatic triple-negative breast cancer. Future Oncol..

[161] Pusztai L., Yau C., Wolf D.M., Han H.S., Du L., Wallace A.M., String-Reasor E., Boughey J.C., Chien A.J., Elias A.D. (2021). Durvalumab with olaparib and paclitaxel for high-risk HER2-negative stage II/III breast cancer: Results from the adaptively randomized I-SPY2 trial. Cancer Cell.

[162] Rizvi N.A., Hellmann M.D., Brahmer J.R., Juergens R.A., Borghaei H., Gettinger S., Chow L.Q., Gerber D.E., Laurie S.A., Goldman J.W. (2016). Nivolumab in Combination with Platinum-Based Doublet Chemotherapy for First-Line Treatment of Advanced Non-Small-Cell Lung Cancer. J. Clin. Oncol. Off. J. Am. Soc. Clin. Oncol..

[163] Langer C.J., Gadgeel S.M., Borghaei H., Papadimitrakopoulou V.A., Patnaik A., Powell S.F., Gentzler R.D., Martins R.G., Stevenson J.P., Jalal S.I. (2016). Carboplatin and pemetrexed with or without pembrolizumab for advanced, non-squamous non-small-cell lung cancer: A randomised, phase 2 cohort of the open-label KEYNOTE-021 study. Lancet Oncol..

[164] Carter C., Caroen S., Oronsky B., Quinn M.F., Williams J., Brzezniak C.E. (2020). Phase I pilot study of RRx-001 + nivolumab in patients with traditionally non-checkpoint inhibitor-responsive cancers (PRIMETIME). J. Clin. Oncol..

[165] Iyer S.P., Xu J., Becnel M.R., Nair R., Steiner R., Feng L., Lee H.J., Strati P., Ahmed S., Parmar S. (2020). A Phase II Study of Pembrolizumab in Combination with Romidepsin Demonstrates Durable Responses in Relapsed or Refractory T-Cell Lymphoma (TCL). Blood.

[166] Segal N.H., Infante J.R., Sanborn R.E., Gibney G.T., Lawrence D.P., Rizvi N., Leidner R., Gajewski T.F., Bertino E., Sharfman W.H. (2016). 1086P—Safety of the natural killer (NK) cell-targeted anti-KIR antibody, lirilumab (liri), in combination with nivolumab (nivo) or ipilimumab (ipi) in two phase 1 studies in advanced refractory solid tumors. Ann. Oncol..

[167] Hanna G.J., O’Neill A., Shin K.Y., Wong K., Jo V.Y., Quinn C.T., Cutler J.M., Flynn M., Lizotte P.H., Annino D.J. (2022). Neoadjuvant and Adjuvant Nivolumab and Lirilumab in Patients with Recurrent, Resectable Squamous Cell Carcinoma of the Head and Neck. Clin. Cancer Res. Off. J. Am. Assoc. Cancer Res..

[168] Duerinck J., Schwarze J.K., Awada G., Tijtgat J., Vaeyens F., Bertels C., Geens W., Klein S., Seynaeve L., Cras L. (2021). Intracerebral administration of CTLA-4 and PD-1 immune checkpoint blocking monoclonal antibodies in patients with recurrent glioblastoma: A phase I clinical trial. J. Immunother. Cancer.

[169] Silk A.W., O’Day S.J., Kaufman H.L., Bryan J., Norrell J.T., Imbergamo C., Portal D., Zambrano-Acosta E., Palmeri M., Fein S. (2021). Abstract CT139: Intratumoral oncolytic virus V937 in combination with pembrolizumab (pembro) in patients (pts) with advanced melanoma: Updated results from the phase 1b CAPRA study. Cancer Res..

[170] Pelster M.S., Amaria R.N. (2019). Combined targeted therapy and immunotherapy in melanoma: A review of the impact on the tumor microenvironment and outcomes of early clinical trials. Ther. Adv. Med. Oncol..

[171] Ribas A., Hodi F.S., Callahan M., Konto C., Wolchok J. (2013). Hepatotoxicity with combination of vemurafenib and ipilimumab. N. Engl. J. Med..

[172] Li H., Xiao Y., Li Q., Yao J., Yuan X., Zhang Y., Yin X., Saito Y., Fan H., Li P. (2022). The allergy mediator histamine confers resistance to immunotherapy in cancer patients via activation of the macrophage histamine receptor H1. Cancer Cell.

[173] Chou C., Zhang X., Krishna C., Nixon B.G., Dadi S., Capistrano K.J., Kansler E.R., Steele M., Han J., Shyu A. (2022). Programme of self-reactive innate-like T cell-mediated cancer immunity. Nature.

[174] Benci J.L., Johnson L.R., Choa R., Xu Y., Qiu J., Zhou Z., Xu B., Ye D., Nathanson K.L., June C.H. (2019). Opposing Functions of Interferon Coordinate Adaptive and Innate Immune Responses to Cancer Immune Checkpoint Blockade. Cell.

[175] Zaretsky J.M., Garcia-Diaz A., Shin D.S., Escuin-Ordinas H., Hugo W., Hu-Lieskovan S., Torrejon D.Y., Abril-Rodriguez G., Sandoval S., Barthly L. (2016). Mutations Associated with Acquired Resistance to PD-1 Blockade in Melanoma. N. Engl. J. Med..

[176] Baruch E.N., Youngster I., Ben-Betzalel G., Ortenberg R., Lahat A., Katz L., Adler K., Dick-Necula D., Raskin S., Bloch N. (2021). Fecal microbiota transplant promotes response in immunotherapy-refractory melanoma patients. Science.

[177] Davar D., Dzutsev A.K., McCulloch J.A., Rodrigues R.R., Chauvin J.M., Morrison R.M., Deblasio R.N., Menna C., Ding Q., Pagliano O. (2021). Fecal microbiota transplant overcomes resistance to anti-PD-1 therapy in melanoma patients. Science.

[178] Gupta J., Safdari H.A., Hoque M. (2021). Nanoparticle mediated cancer immunotherapy. Semin. Cancer Biol..

[179] Nakamura T., Sato T., Endo R., Sasaki S., Takahashi N., Sato Y., Hyodo M., Hayakawa Y., Harashima H. (2021). STING agonist loaded lipid nanoparticles overcome anti-PD-1 resistance in melanoma lung metastasis via NK cell activation. J. Immunother. Cancer.

[180] Li X., Khorsandi S., Wang Y., Santelli J., Huntoon K., Nguyen N., Yang M., Lee D., Lu Y., Gao R. (2022). Cancer immunotherapy based on image-guided STING activation by nucleotide nanocomplex-decorated ultrasound microbubbles. Nat. Nanotechnol..

[181] Buss C.G., Bhatia S.N. (2020). Nanoparticle delivery of immunostimulatory oligonucleotides enhances response to checkpoint inhibitor therapeutics. Proc. Natl. Acad. Sci. USA.

[182] Yang G., Xu L., Chao Y., Xu J., Sun X., Wu Y., Peng R., Liu Z. (2017). Hollow MnO(2) as a tumor-microenvironment-responsive biodegradable nano-platform for combination therapy favoring antitumor immune responses. Nat. Commun..

[183] de Titta A., Ballester M., Julier Z., Nembrini C., Jeanbart L., van der Vlies A.J., Swartz M.A., Hubbell J.A. (2013). Nanoparticle conjugation of CpG enhances adjuvancy for cellular immunity and memory recall at low dose. Proc. Natl. Acad. Sci. USA.

[184] Rosalia R.A., Silva A.L., Camps M., Allam A., Jiskoot W., van der Burg S.H., Ossendorp F., Oostendorp J. (2013). Efficient ex vivo induction of T cells with potent anti-tumor activity by protein antigen encapsulated in nanoparticles. Cancer Immunol. Immunother..

[185] Smith T.T., Stephan S.B., Moffett H.F., McKnight L.E., Ji W., Reiman D., Bonagofski E., Wohlfahrt M.E., Pillai S.P.S., Stephan M.T. (2017). In situ programming of leukaemia-specific T cells using synthetic DNA nanocarriers. Nat. Nanotechnol..

[186] Gonzalez H., Hagerling C., Werb Z. (2018). Roles of the immune system in cancer: From tumor initiation to metastatic progression. Genes Dev..

[187] Spencer C.N., McQuade J.L., Gopalakrishnan V., McCulloch J.A., Vetizou M., Cogdill A.P., Khan M.A.W., Zhang X., White M.G., Peterson C.B. (2021). Dietary fiber and probiotics influence the gut microbiome and melanoma immunotherapy response. Science.

